# Relationship between eating behaviors and physical activity of preschoolers and their peers: a systematic review

**DOI:** 10.1186/s12966-016-0374-x

**Published:** 2016-04-14

**Authors:** Stéphanie A. Ward, Mathieu F. Bélanger, Denise Donovan, Natalie Carrier

**Affiliations:** Faculty of Medicine and Health Sciences, Université de Sherbrooke, Centre de formation médicale du Nouveau-Brunswick, 100, rue des Aboiteaux, Moncton, NB E1A 3E9 Canada; Department of Family Medicine, Université de Sherbrooke, Centre de formation médicale du Nouveau-Brunswick, Vitalité Health Network, Moncton, NB E1A 3E9 Canada; Department of Community Health Sciences, Université de Sherbrooke, Centre de formation médicale du Nouveau-Brunswick, Moncton, NB E1A 3E9 Canada; École des sciences des aliments, de nutrition et d’études familiales, Université de Moncton, Moncton, NB E1A 3E9 Canada

**Keywords:** Peer influence, Physical activity, Eating behaviors, Preschoolers

## Abstract

**Objectives:**

Children learn by observing and imitating others, meaning that their eating behaviors and physical activity may be influenced by their peers. This paper systematically reviews how preschoolers’ eating behaviors and physical activity relate to their peers’ behaviors, and discusses avenues for future research.

**Methods:**

Six databases were searched for quantitative, peer-reviewed studies published up to July 2015 reporting on the correlates, predictors or effectiveness of peers on eating behaviors and physical activity in preschoolers. Risk of bias was independently assessed by two evaluators using the Quality Assessment Tool for Quantitative Studies.

**Results:**

Thirteen articles were included: six measured physical activity, and seven assessed eating behaviors. Four of the six physical activity studies reported that children were more active when peers were present, while large peer group size was negatively associated with physical activity in two cross-sectional studies. All nutrition interventions reported that children’s eating behaviors may be influenced by their peers.

**Conclusions:**

Although supported by weak evidence, peers appear to influence children’s eating behaviors and physical activity. However, this influence may be moderated by the number of peers, gender, age and the perceived status of the role models. Future obesity prevention interventions should consider involving peers as agents for positive eating behaviors and physical activity in preschoolers.

## Background

Approximately 12 % of children under the age of 5 living in developed countries had excess weight or obesity in 2010 [1], with prevalence exceeding 20 % in countries such as Canada [2], the United States [3], and Australia [4]. Childhood obesity generally reflects an imbalance between energy intake and expenditure [5, 6], which is modifiable through dietary behaviors and physical activity [6]. These behaviors are learned during childhood and are sustained through adolescence and adulthood [7, 8]. Acquiring healthy eating behaviors and being physically active in early childhood could therefore be a crucial component of obesity prevention.

According to social facilitation theory the presence of others influences behaviors [9]. For example, it has been shown that adults eat more in the presence of others than when alone, especially when others are familiar [10, 11]. Studies in non-athlete adults have also reported increased exercise intensity while walking and cycling when in the presence of an unfamiliar single peer or group of peers [12, 13]. Effects of social facilitation on eating behavior of youth are not as clear [14, 15]. Although Savly et al. (2007) found that overweight school-aged children ate more when they were alone than when they were in the presence of other children, they observed that normal-weight children ate more with others than they did when alone [15]. The literature on physical activity also supports the concept of social facilitation among youth, reporting they tend to be more physically active when in the presence of peers and friends [16–18]. Observational learning theorists suggest that children’s behaviors are influenced by the behaviors of those in their entourage [19]. Accordingly, individuals mimic behaviors they perceive as important and as likely to lead to rewarding outcomes [20]. Although studies have consistently shown that modeling has a strong effect on eating behaviors and physical activity of adults, school-aged children and adolescents [9, 21–28], very little has been reported for preschoolers.

Preschoolers’ behaviors are influenced by those of their parents [29–32]. However, since the preschool years represent the first stage of life where many children start separating from their home and become exposed to new environments (e.g. childcare centres) and to new sources of social influence (i.e. peers, educators), it is possible that preschoolers modify their behaviors in response to observed norms, regulations and expectations of educators and other children [33]. It has been suggested that children begin to show an appreciation for normative behavior as they progress through the preschool years [34], and that preschoolers are preoccupied by social inclusion [35]. Moreover, preschoolers are thought to be particularly likely to reproduce behaviors of those they perceive as similar to themselves [19]. Hence, peers potentially represent role models for the development of healthy eating behaviors and physical activity among preschoolers. As such, it may also be possible to involve peers in health promoting interventions aiming at reaching a large number of children. To help document the influence of peers and inform potential interventions, this paper systematically analyses quantitative studies published up to July 2015 that have examined the relationship between preschoolers’ eating behaviors and physical activity, and those of their peers. Gaps in the literature in this area are identified and avenues for future research are discussed.

## Methods

### Protocol and registration

This study followed the procedures for systematic review reporting as described by the Preferred Reporting Items for Systematic Reviews and Meta-Analyses (PRISMA) recommendations [36], used the same methods as the ones we described in details elsewhere [37], and was registered with the International Prospective Register of Systematic Reviews (PROSPERO) (record #CRD42014015450).

### Search strategy

The search strategy, including the choice of database selection, was developed in collaboration with an experienced medical research librarian. The computerized literature search was completed in July 2015 in Science Direct, PsychInfo, PubMed, Medline, ERIC, SportDiscus and CINAHL. The search strategy included four groups of keywords: peers (e.g. “peer group”, “peer influence”,”peer model*, “peer effect”), physical activity (e.g. “physical activity”, “exercise”, “sport”, “movement skills”, “motor activity”), eating behavior (e.g. “eating behavior”, “food consumption”, “food intake”, “food preference”, “food choice”, “food neophobia”, “food habits”), and population (e.g. “preschool child”, “young child”, “child”). Where possible, limits on language (English and French) and age (preschool child) were used. Reference lists were also reviewed to identify and retrieve potentially eligible studies.

### Eligibility criteria

All types of quantitative studies published in either English or French found in peer-reviewed journals were included, in order to ensure a comprehensive review of the existing literature. Non-randomized and observational-type studies were included as they can provide impetus for future randomized controlled trials (RCT) [38]. No publication date restrictions were used in any of the databases.

Eligible studies were those whose subjects were preschoolers without medical disabilities or disorders, between 2 and 5 years of age, and those which had separate analyses for children in that age group. We defined peers as friends, or playmates who were younger or older, familiar or unfamiliar to the target child. Since siblings may influence behaviors differently than those of peers, studies focusing on siblings were excluded.

All objective and subjective measures of physical activity and eating behaviors were considered for this review. Physical activity variables included frequency or duration of physical activity at different intensity levels (i.e. sedentary, light, moderate or vigorous). Nutrition variables included the amount or type of food consumed (i.e. increased fruit and vegetable intake), and eating behaviors, such as reluctance towards certain foods.

### Study selection and data collection process

After removal of duplicates, the first author checked the titles and abstracts of identified studies against the inclusion criteria. The full texts of all potentially eligible studies were reviewed by two authors, that is, by the first author and by one of the other three co-authors. Data from all included studies were entered into an electronic study-specific data extraction sheet by the first author. The other authors each extracted data from one-third of the publications. At all stages of this review, disagreements were resolved through discussion among authors.

### Quality assessment and risk of bias

All included studies were assessed for quality and risk of bias using the Effective Public Health Practice Project Quality Assessment Tool for Quantitative Studies [39]. This tool was chosen because of its ability to assess the quality of various quantitative study designs relating to public health topics. Risk of bias was assessed at the study level for six components: (i) selection bias; (ii) study design; (iii) confounders; (iv) blinding; (v) data collection method; and (vi) withdrawals and dropouts. Each of these six components were rated on a three point scale as strong, moderate or weak, leading to an overall methodological quality rating score of strong (no weak individual scale rating), moderate (one weak individual scale rating) or low (two or more weak individual scale ratings) [39]. Each study was reviewed for quality and bias by two authors.

### Strength of evidence

As suggested by Harbour and Miller (2001) as well as the Department for International Development, observational and intervention-type studies can greatly contribute to the overall strength of evidence in domains such as behavioural research [40, 41]. Following their recommendations, we based strength of evidence on the quality and quantity of studies and on the consistency of the results using a rating system used in previous studies [42, 43]. Evidence was considered as strong if at least two RCTs of high quality showed consistent results. Moderate evidence was concluded if at least one RCT of high quality, and at least one RCT of moderate or low quality or one non-randomized controlled trial of high quality showed consistent results. Evidence was considered as weak if there was only one RCT of high quality, or multiple moderate to low quality RCT and non-randomized controlled trials of high, moderate or low quality, all showing consistent results. Finally, insufficient evidence was concluded if there was only one low or moderate quality RCT or one high, moderate or low quality non-randomized controlled trial, or if contradictory outcomes were reported. Consistency of results was defined as significant results in the same direction, reported in at least two-thirds of the studies [42]. This meant that regardless of study design, methodology or measurement tools, results were considered as consistent if the relationship between the exposure and the outcome for a given construct (physical activity or eating behaviours) were similar (positive or negative).

### Data synthesis and statistical analyses

Extracted variables from all the included studies were reported using a standardized form and included: (i) study characteristics (e.g. author names, year, country of origin); (ii) study design; (iii) sample characteristics; (iv) study setting; (v) description and aim of the study; (vi) outcome measurement tools; and (vii) study results. When available, means or odd ratios (OR), p-values and confidence intervals (CI) are presented. Scores for each component of the quality assessment and the overall quality score are presented for each study.

## Results

### Study selection

**Fig. 1:**
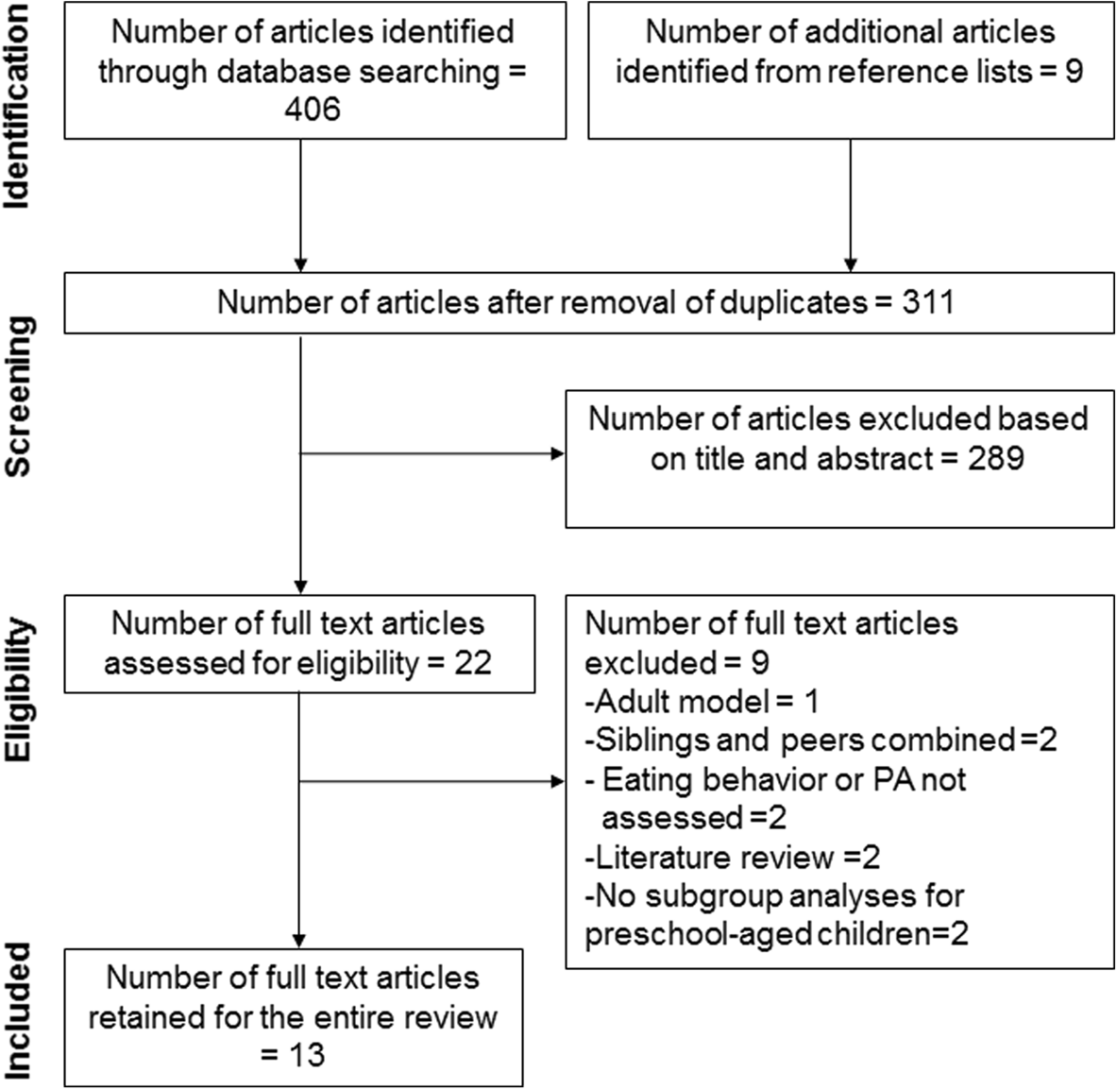
PRISMA flow diagram of study selection process

The study selection process, including the reasons for excluding studies, is summarized in Fig. 1. Of the 311 studies identified, 22 were retained after review of their titles and abstracts. Of these, 13 were retained after the full-text review.

### Study characteristics

Of the thirteen studies retained, six assessed the relationship between peers and preschoolers’ physical activity [44–49], while seven assessed the influence of peers on children’s eating behaviors (Table 1) [50–56]. Of those examining physical activity, two were RCTs [44, 47], one was a non-randomized controlled trial [42] and three were cross-sectional [45, 47, 48]. Of those examining eating behaviours, one was a RCT [50], three were non-randomized controlled trials [51, 52, 54] and three were pre-post studies [50, 51, 55]. The largest sample sizes were seen in the three cross-sectional studies in Finland (*n* = 892 children), the United States (*n* = 476 children), and the Netherlands (*n* = 175 children). Sample sizes in the ten experimental studies were much smaller and ranged from 14 to 69 children. Although outcomes were measured mostly in childcare centres (also referred to as preschools, nurseries or kindergartens), two physical activity interventions were conducted in laboratories [44, 49] and one in a research trailer [46].

**Table 1:** Characteristics of included studies

Study (country)	Study design	Sample	Setting	Description/Aim	Outcome measurement tools	Results
Physical activity						
Barkley et al. 2014 (USA) [44]	Cross-over controlled trial	20 children (50 % girls) 3–6 years	Laboratory	Children’s PA was assessed during 30 minute sessions under two social conditions: while playing alone and with a friend. Aim: To assess the effect of the presence of a friend on amount and intensity of PA	PA: Accelerometer Sedentary activity: Direct observation by research personnel	Children had 54 % greater (*P* < .02) average accelerometer counts during the friend condition (μ = 2629, SD = 1080 or 5.7 METs) than during the alone condition (mean = 1707, SD = 1009 or 4.5 METs).
Brown et al., 2009 (USA) [45]	Cross-sectional	476 children (51 % boys) 3–5 years; 55 % African Americans	Preschool	Children’ PA and context was observed and recorded during indoor and outdoor activities at preschool. Aim: To determine which contextual conditions were predictors of PA of children during outdoor play.	PA and social environment: Observational System for Recording Activity in children (OSRAC-P)	Compared to outdoor activities with an adult present, MVPA was 3.55 times more likely if children were alone, 2.29 times more likely when one-to-one with another peer, and 2.04 times more likely when in a group of peers. Non-sedentary PA was 2.77 times more likely when children were alone, 1.53 times more likely when one-to-one with a peer and 1.48 times more likely when with two or more peers without adults, compared to activities with an adult present.
Eaton & Keats, 1982 (Canada) [46]	Cross-over clustered-RCT	69 children (27 girls) Mean age = 51.1 months (4.3 years);	Mobile research trailer	Children were randomly assigned to same-sex triads and visited the play setting twice, once alone and the other with peers. Aim: To test whether girls’ and boys’ PA is influenced by same-sex peers and if they are influenced differently	PA: Accelerometer	Girls and boys were more active in triads than alone (girls: μ = 3.43, SD = .19 vs μ = 2.80, SD = .24; boys: μ3.56, SD = .24 vs μ = 3.15, SD = .23). Peer presence did not influence boys and girls differently.
Gubbels et al., 2011 (Netherlands) [47]	Cross-sectional	175 children 2–3 years; mean age = 2.6 years	Childcare centre	Children’s PA was observed at childcare centres and aspects of the environment were assessed. Aim : To examine the association between the social and physical childcare environment and PA of children	PA and social environment: OSRAC-P	Indoor prompts by peers were not associated with PA (*P* = .966). Negative prompts by peers had a positive effect in boys (*P* < .05) but not in girls (*P* > .05). Positive peer prompts had a stronger effect in boys than girls (both *P*’s < .01). Larger group size of peers was associated with lower PA both indoors (*P* < .001) and outdoors (*P* = .015). 3 year-olds’ PA was negatively associated by one (*P* < .05) or more (*P* < .001) peers present. 2 year-olds’ PA was not associated with group size (*P* > .05).
Lehto et al., 2012 (Finland) [48]	Cross-sectional	892 children (51 % boys) Mean age = 4.7 years (SD = 1.313)	Childcare centre	Children’s PA level and nearest peer contact was observed during childcare hours. Aim: To investigate the association between peer relations and PA	PA: Direct observation by research personnel Personality and skills: Evaluated by teachers using a 5 point scale	Children who were physically active sought each other’s company (*P* < .001). When 3–5 year old children interacted with a group of children, their percentage of high PA was the highest (18.8 % of the time), while interaction with one child was second highest (12.4 %). When children were more withdrawn from other children, their PA tended to be lower (*P* = .006). Children tended to be less physically active when in the presence of a more independent and self-directed peer (*P* < .001).
Schwarz, 1972 (USA) [49]	RCT	57 children (22 boys) 4 years	Laboratory	Children’s mobility was videotaped for 5 minutes in an unfamiliar room under one of three conditions: with a close friend, with an unfamiliar peer or alone. Aim: To examine the influence of a peer in an unfamiliar situation on distress of preschoolers.	Children’s mobility: Direct observation with videotape Distress: rated by teachers from videotape recordings	Mobility was greater in the friend condition than in the stranger or alone conditions (*P* < .05). No significant differences were shown for motility between stranger and alone conditions.
Eating behaviors						
Birch, 1980 (USA) [50]	Pre-, post study	39 children (20 girls); 87 % Caucasian; middle-class 2.11–4.10 years; Median age = 3.10	Nursery	Target children who preferred vegetable A to B were seated with 3 or 4 peers with opposite preferences. Children were served their preferred and non-preferred vegetable and asked to choose one. Aim: To investigate peers’ short and long-term influence on preschoolers’ food choices and eating behaviors.	Food preference rating: Assessment of taste preference of nine vegetables using a “Faces” Likert scale depicting a food as good, bad or ok. Amount of food consumed: Observer recorded the number of tablespoons served and plate waste was recorded	Target children who chose their preferred food on day 1 chose their non-preferred food on day 4 (*P* < .05). Target children made significantly more choices of their non-preferred food than their peers (*P* < 0.001). Younger children were more influenced by their peer than the older children (*P* < .05). Post-influence assessment found that 12 of the 17 target children increased their preference for the non-preferred food (median increase of 2.5 positions), while less than half of the peers did so.
Duncker, 1938 (England) [51]	Pre, post-design	Study 1: 2.8–5.2 years; urban, low-income Study 2: 31 children Mean age = 4.5 years	Nursery	Children were selected as either a predecessor or a successor and had opposite preferences for six food items. Pairs of the food items were presented and both children chose the food they preferred. The predecessor made his choice in front of the successor. A story was told to children about how the heroes liked Maple sugar than Hemlock bark. Modified sugars were used to represent the foods in the story and children were asked to pick which one they preferred. Aim: To examine the influence of peers and age of peers on food choices of children	Food choices: Direct observation of the food item chosen	Children made more identical choices (81 %) in the experimental condition than in the control condition (25.6 %). Younger children made more identical choices when the predecessor was older (26/28) than when the predecessor was younger (14/24). When the predecessor has a high degree of prestige or friendship with the successor, the successor made identical choices for all food items (100 %).
Greenhalgh et al., 2009 (Wales) [52]	RCT	49 children 3–4 years	Nursery	Children were randomized into either Group A, B or C and received a novel food on four snack occasions. Group A received positive modeling of the novel food on the first and third occasions, and were alone on the second and fourth occasions. Group B received negative modeling on the first occasion, positive modeling on the third, and were alone on the second and fourth occasions. Group C was alone at all four occasions. Aim: To determine the influence of peer modeling on young children’s consumption or rejection of a novel food.	Amount of food consumed: Visual estimation of plate waste using a five-point scale	Children ate more of the target food when exposed to positive peer modeling than when exposed to negative modeling (Phase 1: *P* < .001; Phase 2: *P* < .001), and ate less of the target food when exposed to negative modeling than when peers were absent (Phase 1: *P* = .001; Phase 2: *P* = .009) . The mean difference between the negative modeling group and the control group (μ = 43.75 %) was greater than the mean difference between the positive modeling group and the control group (μ =16.25 %).
Hendy & Raudenbush, 2000 (USA) [53]	Controlled trial	14 children (6 boys) Mean age = 51.4 months (SD = 11.0)	Childcare centre	Children’s number of bites of new food was videotaped across five meals. Three new foods were presented with enthusiastic teacher modeling, enthusiastic peer modeling, or simple exposure (no modeling). Delayed food acceptance was gathered one month later. Aim: To compare the effectiveness of teacher modeling and peer modeling on acceptance of new food and whether peer modeling modified the effects of teacher modeling.	Amount of food eaten: Direct observation of number of bites eaten, recorded by researchers Food acceptance: preference ratings were obtained with a “Faces” Likert scale depicting food as good, bad or ok	Boys accepted new foods equally under all three modeling conditions (*P* < .43), while girls accepted new foods most when modeled by peers *P* < .03). With trained peer models, girls’ number of bites increased across the meals. Immediate acceptance and delayed acceptance of peer modeled foods was greater for girls (*P* < .04) than boys (*P* < .002). Enthusiastic teacher modeling was ineffective if competing peer models were present.
Hendy, 2002 (USA) [54]	Controlled trial	38 children (50 % boys) 3–6 years; mean age = 54.7 months (SD = 7.9); 86.8 % Caucasian; rural, low-income	Preschool	Peer models were trained by preschool teachers. Three novel foods were presented to children during five lunch meals (3 baseline meals, 2 modeling meals). Each food was assigned to either no model, girl model or boy model conditions. Delayed food preference was assessed one month later. Aim: To examine the effectiveness of trained peer models to encourage food acceptance in children during preschool lunch, and one month later.	Amount of food consumed: Direct observation of number of bites taken, recorded by research assistants Food preference rating: Assessment of taste preference of the three novel foods using a “Faces” Likert scale depicting a food as good, bad or ok.	Same-gender models were no more effective than opposite-gender models in increasing food acceptance (*P* = .768). Girl models were more effective than boy models to increase food acceptance of children of either gender from baseline to modeled meals (*P* = 0.014). For target children, no significant differences were found for delayed food preference ratings (*P* = .731) or number of bites (*P* = .557) from the modeling condition to the one month assessment.
Lumeng & Hillman, 2007 (USA) [55]	Pre-, post study	54 children (68 % boys) 2.5–6.5 years; mean age = 4.2 years (SD = 1.1); 74 % Caucasian	Preschool	Children ate a standardized snack in a group of three and nine children. Consumption was videotaped. Aim: To determine the effect of group size on children’s food consumption	Amount of food eaten: Number of crackers eaten recorded on videotape	Children ate slightly more when eating in larger groups, than when eating in smaller groups (*P* = .03). During short snacks, there was no effect of group size on amount eaten (*P* = .42). During long snacks, large group size increased the amount eaten by 30 %.
Marinho, 1942 (Brazil) [56]	Controlled trial	66 children 4–6 years	Kindergarten	Children were divided into groups according to their food preference (predominant and indefinite taste) and subdivided into experimental and control groups. A peer was chosen as the leader and chose the food that the target child disliked. The target child was then asked to choose one of the foods. After eliminating peer influence, children’s isolated choices were assessed over 5 weeks and 2 weeks one year later. Aim: To determine if a leader causes lasting modifications of a child’s original taste preference.	Food choice and type of leadership: Direct observation by researcher	50 % of children with predominant taste modified their original taste. After-effects were observed for 48.9 % in the first four choices after the experiment and 16.7 % showed after-effects one year later. 100 % of children with originally indefinite taste modified their choice during the experiment. All but four showed after-effects one year later. Children modified their choice when the leader was socially agreeable but not when the leader was domineering.

*RCT* randomized-controlled trial

The physical activity-related studies assessed the relationship between peer presence or the number of peers present, and preschoolers’ physical activity. One cross-sectional study also examined the association between peer prompts and physical activity levels [47], while a second cross-sectional study examined the relationship between personality traits of peers and physical activity levels [48]. In all six studies, physical activity levels were quantified using either direct observation [44, 45, 47–49] or accelerometers [44, 46].

Most of the eating behavior-related studies examined the influence of peers’ food preferences or choices on children’s food preferences, choices or consumption. Other aspects studied included the influence of peer modeling [52–54], as well as the age [51] and the status (e.g. popularity, leader) of the peer model [51, 56], and group size [55] on preschoolers’ consumption, acceptance/rejection, or choice of food. All studies that assessed children’s food consumption or choices used either direct observation [50–56] or plate waste [50]. Food preference or acceptance was assessed via “Faces” Likert scales in three studies [50, 53, 54].

### Methodological quality assessment

Of the six physical activity-related studies, two received moderate quality score ratings [44, 46] and four received low quality score ratings [45, 47–49] (Table 2). Two of the seven nutrition-related papers received moderate ratings [52, 53], and the remaining five were scored as low [50, 51, 54–56]. Regardless of the study design, low ratings were mostly attributable to the likelihood of selection bias; causes included the possibility of poor representation of the target population and low response rates. Other methodological limitations were lack of information on the validity and reliability of data collection tools or methods, and, in the case of the eating behavior-related papers, the missing numbers of withdrawals and dropouts.

**Table 2:** Results of quality assessment of studies using the EPHPP^a^ Quality Assessment Tool for Quantitative Studies

Study authors and date	Selection bias	Study design	Confounders	Blinding	Data collection methods	Withdrawals and dropouts	Overall quality score
Physical activity							
Barkley et al., 2014 [44]	Weak	Strong	Strong	Moderate	Strong	Strong	Moderate
Brown et al., 2009 [45]	Weak	Weak	Strong	Moderate	Weak	N/A	Low
Eaton & Keats, 1982 [46]	Weak	Strong	Strong	Moderate	Strong	Strong	Moderate
Gubbels et al., 2011 [47]	Moderate	Weak	Strong	Moderate	Weak	N/A	Low
Lehto et al., 2012 [48]	Weak	Weak	Strong	Moderate	Weak	N/A	Low
Schwarz, 1972 [49]	Weak	Strong	Weak	Strong	Weak	Strong	Low
Eating behaviors							
Birch, 1980 [50]	Weak	Moderate	Weak	Moderate	Weak	Strong	Low
Duncker, 1938 [51]	Weak	Moderate	Weak	Weak	Weak	Weak	Low
Greenhalgh et al., 2009 [52]	Moderate	Strong	Weak	Moderate	Strong	Strong	Moderate
Hendy & Raudenbush, 2000 [53]	Moderate	Strong	Strong	Moderate	Weak	Moderate	Moderate
Hendy, 2002 [54]	Moderate	Strong	Strong	Moderate	Weak	Weak	Low
Lumeng & Hillman, 2007 [55]	Weak	Moderate	Strong	Moderate	Weak	Weak	Low
Marinho, 1942 [56]	Weak	Strong	Weak	Moderate	Weak	Weak	Low

^a^EPHPP: Effective Public Health Practice Project

### Relationship between peers and children’s physical activity

Four of six studies reported a positive relationship between peer presence or number of peers present and children’s physical activity levels [44, 46, 48, 49]. These studies concluded that, compared to being alone, children were more physically active when one or more peers were present. For example, a moderate quality cross-over controlled trial by Barkley et al. (2014) reported that children had 54 % greater accelerometer counts when in the presence of a friend, compared to being alone [44]. Similar results were found in a RCT among 69 Canadian preschoolers, where children were more active when in the presence of two same-sex peers, compared to being alone [46]. A large cross-sectional study also reported that children spent the greatest percentage of time in physical activity when in the presence of a group of peers, while the presence of only one peer accounted for the second highest percentage of time spent in physical activity [48]. Despite these findings, two low quality cross-sectional studies found that a larger peer group size was associated with lower levels of physical activity [45, 47]. One of these suggested that children’s physical activity levels is dependent on who they are with as activity was lowest in the presence of educators, higher with two or more peers, higher again with one peer and highest when alone [45]. A second cross-sectional study also found that larger group size was linked to lower levels of physical activity, both outdoor and indoor, but that this relationship was only seen among three year-old children and not among two year-olds [47].

The familiarity and the personality of the peers could be moderating factors. For example, Schwarz et al. (1972) found that children had higher levels of physical activity when in the presence of a friend compared to a stranger, and that there were no differences in physical activity between being in the presence of a stranger and being alone [49]. Another cross-sectional study found that children spent the highest percentage of time in physical activity when with interactive peers and were less physically active with independent, self-directed peers [48].

One RCT found that girls’ and boys’ physical activity levels were higher when in the presence of two same-sex peers, compared to being alone, but that the difference in activity levels between the alone and triad conditions were similar between sexes [46]. On the contrary, one cross-sectional study found that prompting by peers to be physically active was linked to higher physical activity levels in boys than in girls [47]. The latter study also reported that prompts that discouraged physical activity outdoors (i.e. short verbal messages) were linked to higher physical activity levels in boys but not in girls, and that boys responded more positively to prompts that promoted physical activity than girls [47].

Based on the strength of evidence evaluation, there is currently weak evidence to suggest that peers influence preschoolers’ physical activity levels.

### Relationship between peers and children’s eating behaviors

Results suggest that children’s food choices, preferences and consumption are associated with peers in various ways. Two studies found that when peers or a specific peer model chose a child’s non-preferred food, preference for that food increased [50, 56]. When looking specifically at peer modeling, positive peer modeling was shown to be more effective than no modeling in increasing the intake of a target food, and that negative peer modeling could decrease it [52]. The effect of peer modeling may be moderated by gender. For example, Hendy and Raudenbush (2000) found that girls took more bites and increased their acceptance of new foods when in the presence of peer models, compared to boys, who accepted new foods regardless of whether they were exposed to enthusiastic educator modeling, peer modeling, or no modeling [53]. Hendy (2002) also found that girls were better role models to increase food acceptance in both genders, and that same gender peer modeling was not more effective [54]. Peers may also have a different impact depending on their age [50, 51] and how they are perceived by other children [51, 56]. For example, two studies reported that younger children were more influenced by older children [50, 51], and that children made similar choices to peers with whom they were friends or who were generally liked by their peers, and who had higher prestige [51, 56], compared to peer models who were domineering [56]. The order of access to food choice may be important: in one study, children often chose the same food as the previous child chose [51]. Finally, one study found that children ate more food at snack time when in a large group (nine children) compared to when in a smaller group (three children) [55].

Despite the lack of high quality studies, consistent results were reported across all nutrition-related studies, suggesting that there is weak evidence that peers influence preschoolers’ eating behaviors.

## Discussion

This systematic review provides weak evidence that peers may act as role models for children’s eating behaviors and physical activity, which aligns with the theory of social facilitation and observational learning. Results also indicate that the influence of peers may be moderated by a number of peer-level variables, such as gender, age and the perceived status of the role models. There exists no simple solution to combating and preventing childhood obesity. Targeting peers in public health interventions will not solve the epidemic, however it could contribute to its improvement. Although the evidence to date is weak, due to the dearth of high quality studies, results from this review suggest that positively influencing the behaviors of some preschoolers has the potential to affect many others. From this perspective, based on theoretical constructs [57, 58], and from findings among adults that social norms are important determinants of physical activity and healthy eating [59–62], it appears important to develop and test interventions aimed at modifying social norms relating to these behaviors among preschoolers. By extension, promoting opportunities for children to be exposed to situations where there are peers who display desirable eating behaviors or physical activity, could contribute to reducing overall obesity rates. Further research based on high quality study designs, such as RCTs, with larger sample sizes and which use valid and reliable measurement tools are needed in order to strengthen the evidence that peers are key actors in physical activity and healthy eating promotion among young children.

### Physical activity

Notwithstanding being supported by weak evidence, preschoolers seem to have greater physical activity levels when in the presence of peers. This agrees with the social facilitation theory and other studies that found that older children are more physically active when in the presence of friends or peers [9, 16–18, 63]. However, what is observed may be the effect of active children seeking other physically active children [48], making it likely that these groups of children motivate each other to sustain higher levels of physical activity intensity. Although peers are conducive to physical activity, too many children in one group may impede activity. They may limit the space and equipment, thereby reducing the opportunity to be physically active [64]. This could explain why, in one study, larger groups of children were less likely to be active indoors or outdoors, and why older children, who need more space and play equipment to be active [65], are more influenced by the number of peers [47]. Future studies should investigate the ideal group size which would encourage higher levels of physical activity.

Our results suggest that how well children know each other and children’s personalities, both influence their participation in physical activity [48, 49]. This coincides with the theory of observational learning, which suggests that individuals mimic the behaviors of those whom they perceive as similar to themselves [19]. Children who are introverted or who may not have close friendships with their peers, may feel uncomfortable or excluded, and revert to solitary, low intensity physical activities. Small groups of friends or of children who have similar personal traits may have a greater influence on the physical activity of their members than larger, more diverse groups of children.

Little is still known about whether or not sex is a moderator of peer influence, and about how boys and girls could be influenced differently. Although peer group size influenced boys and girls similarly in one experimental study [46], a cross-sectional study found that boys responded more positively to peer prompting with short verbal messages than did girls [47]. Since boys tend to be more active than girls [66], they may also be more easily influenced than girls to be physically active.

Future studies should generate evidence on the long term effects of peers’ influence on children’s physical activity levels and how subgroups of children (i.e. younger versus older, inactive versus active children, extraverted versus introverted) influence each other’s physical activity levels.

### Eating behaviors

Similar to our findings for physical activity, there is weak evidence that peers influence eating behaviours of preschoolers. Results from this review suggest that children’s eating behaviors may be influenced by their peers’ food choices, preferences and modeling. Our results are similar to those reported in another recent systematic review that included children (1 to 12 years of age), adolescents and adults [67], which found that social influence, particularly modeling, was a strong determinant of individuals’ food intake. The authors of the latter review also suggest that there is strong evidence that modeling increases when individuals perceive themselves to be similar to the model and that the effect of social modeling is partially mediated through mimicry [67]. These are promising findings, especially for childcare educators who wish to encourage children to try new foods.

There is a need to explore whether there are differences between the modeling effect of boys and girls. One study suggested that same-sex peer models were no more effective than opposite sex peer models, but that girls could be more effective role models than boys for improving food preferences [54]. This may concur with the finding that peer models are more effective when they are sociable [56], are well perceived by others, or maintain friendships [51], as young girls tend to be less aggressive and more compliant and socially-involved than boys [68]. Girls also seem to be more influenced by peer models than boys [53] which may be due to peer acceptance being more important to girls than boys [69].

The age of the peer model may also be an important factor to consider. For example, one study concluded that younger children were more influenced by older peers compared to younger ones [51]. In line with the theory of observational learning [19], younger children may look up to older children and mimic their behaviors. Therefore, grouping older and younger children at snack and meal times may encourage younger children to try and eat new or less-preferred foods if the older child models the desired behavior. Since children were also shown to eat slightly more when eating in the presence of a large group [55], having large groups of children of different ages may encourage preschoolers to eat greater amounts of healthy foods. As with physical activity, there is a need to better understand how peers influence children’s eating behaviors in the long term. Further, since most studies looked at positive peer modeling to promote healthy eating behaviors, evidence is lacking on the effects of negative modeling by peers.

### Methodological quality of the studies

Regardless of their design, the quality of the studies was mainly affected by the likelihood that participants were unrepresentative of the target population due to the use of convenience samples and lack of reporting of the response rates. These limitations are common in epidemiological studies. Study participation rates have decreased in recent years [70], and information on study participation is seldom provided in peer-reviewed papers [71]. Information on the validity of data collection methods and tools was also lacking. Although most used objective measurement tools, only three of the thirteen studies included in this review reported the validity of these tools. The use of valid tools for assessing physical activity and nutrition-related outcomes have been shown to be particularly challenging in epidemiological studies because they are often impractical and costly to use in large populations [72]. Lack of reporting or high rates of withdrawals or dropouts was also common in the nutrition-related studies.

A lack of high quality studies precluded the attainment of strong ratings for the strength of evidence in this review. Strength of evidence was based on the quality (i.e. design type and risk of bias) and quantity of studies, and on the consistency of results among studies for a given construct. Given that one third of the physical activity studies included in this review were RCTs, the quantity of studies was not primarily responsible for the final rating of evidence as weak. Higher quality RCTs are therefore needed to strengthen the evidence related to peers’ influence on physical activity.

Although all of the nutrition-related studies included an intervention component (i.e. RCT, controlled trials and pre-post studies), only one was a RCT, which diminished the strength of evidence for this construct. In addition, the majority of studies included were of low quality. Therefore, in order to strengthen the evidence, high-quality RCTs must be conducted in the future. Specifically, RCTs that use larger sample sizes, and valid and reliable measurement tools are needed to improve the overall evidence that peers influence children’s eating behaviours and physical activity.

### Limitations

We acknowledge certain limitations of this review. The heterogeneity of the study designs, outcomes and measurement tools did not allow meta-analysis. Also, as in any systematic review, eligible studies may have been missed by our search strategy. Furthermore, since most of the studies were conducted in high income countries, results may not be applicable in low to middle income countries. Despite these limitations, strengths of this review include a detailed systematic search strategy developed in collaboration with a health-sciences reference expert, not restricting the publication period, including two researchers at every stage of the review process, and using a widely-used, validated tool for assessing the quality of various types of quantitative study designs.

## Conclusions

This is the first review to systematically analyse empirical evidence on the relationship between peers and preschoolers’ eating behaviors and physical activity. Despite the limited number of high quality studies, results support the concept of social facilitation and observational learning theories suggesting that peers may be role models to the adoption of healthy behaviors in preschoolers. Our results also suggest that this relationship may be moderated by a number of variables, such as age, sex, and the perceived personality of the role models. In order to strengthen this evidence, further RCTs with larger sample sizes which use valid and reliable measurement tools are needed. Nevertheless, current evidence suggests that future obesity prevention interventions aiming at reaching a large number of children should consider involving peers as agents for positive eating behaviors and physical activity in preschoolers.

## Abbreviations

PRISMA: Preferred Reporting Items for Systematic Reviews and Meta-Analyses
PROSPERO: International Prospective Register of Systematic Reviews
RCT: Randomized controlled trial
OR: Odd ratios
CI: Confidence intervals
